# Integrating Morphological, Pathogenic, and Molecular Approaches to Characterize *Fusarium* Root Rot Pathogens of Common Bean in Egypt

**DOI:** 10.3390/cimb47100803

**Published:** 2025-09-29

**Authors:** Taghrid A. Kamel, Manal M. Yasser, Naglaa A. Taha, Dalal Hussien M. Alkhalifah, Marym A. Marzouk, Wael N. Hozzien, Walaa R. Abdelghany

**Affiliations:** 1 Plant Pathology Research Institute, Agricultural Research Center, Giza 12619, Egypt; 2 Botany and Microbiology Department, Faculty of Science, Beni-Suef University, Beni-Suef 62511, Egypt; 3 Department of Biology, College of Science, Princess Nourahbint Abdulrahman University, Riyadh 11671, Saudi Arabia

**Keywords:** *Phaseolus vulgaris*, *Fusarium* root rot, pathogenicity, molecular phylogenetic analysis

## Abstract

*Fusarium* root rot (FRR) is a major disease affecting common bean (*Phaseolus vulgaris* L.) production worldwide. In Egypt, FRR has recently become more prevalent, threatening crop yields. *Fusarium* species are considered the primary causal agents of this disease. To identify the pathogens associated with FRR and evaluate host resistance, *Fusarium* isolates were obtained from diseased common bean plants collected in different Egyptian governorates. Morphological identification, pathogenicity assays on six cultivars (Alpha, Samantha, Giza 6, Giza 12, Cambo, and Nebraska), and molecular identification using *TEF-1α* gene sequencing were conducted. Thirteen isolates exhibited pathogenicity, and five isolates (FP33, FP24, FP26, FP21, and FP11) were classified as highly aggressive. Isolate FP33 caused the highest disease severity, reaching 90% on Giza 6 and 80% on Cambo, while Nebraska showed the highest resistance (30% disease severity). Similarly, FP24 led to 85% and 75% severity on Giza 6 and Cambo, respectively. Nebraska and Giza 12 showed the greatest resistance, while Giza 6 and Cambo were most susceptible. Molecular analysis identified FP33 and FP24 as *F. equiseti*, FP26 and FP21 as *F. oxysporum*, and FP11 as *F. solani*. The study demonstrates the genetic and pathogenic variability among *Fusarium* isolates causing root rot in common bean. Nebraska and Giza 12 were identified as the most resistant cultivars, while Giza 6 and Cambo were highly susceptible. These findings highlight the importance of selecting resistant cultivars and implementing integrated disease management strategies to mitigate FRR in Egypt. The results also contribute valuable data for breeding programs aimed at developing durable resistance. The integration of morphological, molecular, and pathogenicity data provides a framework for future epidemiological studies and sustainable disease management strategies.

## 1. Introduction

Among species in the Phaseolus genus, the common bean (*Phaseolus vulgaris* L.) holds the greatest economic significance, being extensively cultivated and considered the most significant leguminous crop, particularly for direct human consumption. Common bean is a vital nutritional crop, valued for its high protein content, which contributes to its status as one of the most significant pulse crops globally. In addition to protein, it serves as a rich source of dietary fiber, iron, essential vitamins, minerals, and complex carbohydrates [1]. As a result, it is a vital crop for global food security, especially in developing countries, where it contributes to combating malnutrition among millions of smallholder farmers [2]. It accounts for 85% of global bean production [3], with 37.8 million hectares harvested annually, yielding approximately 28.5 million tons in 2023 and feeding over 300 million people [4]. In Egypt, the total cultivated area reaches 28,291 hectares for dry beans, producing around 174,178 tons per year [4], while green beans are grown on 28,390.32 hectares, yielding approximately 2.82 million tons annually [5].

Fungal diseases can lead to severe yield and quality losses, ranging from 20% to 100%, depending on factors such as the specific diseases affecting the crop, the severity of the infections, environmental conditions, and agricultural management practices [6]. These pathogens are also known to affect common beans as foliar and seed-borne diseases [6,7,8]. Despite its significance, common bean cultivation is hindered by numerous challenges, including pests and diseases caused by various bacteria [9,10], fungi [11], nematodes, and insects [12], along with abiotic stresses such as salinity [13], drought [14], and heat [15]. These biotic and abiotic factors can lead to substantial reductions in yield and quality, with losses often reaching up to 60% [14]. *Phaseolus vulgaris* L. plants attacked by numerous pathogens cause various diseases, such as root rot, stem rot, rust, and anthracnose. Among the most damaging diseases is root rot, which is caused by fungi like *Fusarium*, *Pythium*, *Macrophomina*, *Sclerotium*, and *Rhizoctonia* species [16]. Fungal pathogens are responsible for significant yield losses in common bean production, with root rot alone accounting for an estimated 221,000 metric tons of annual losses in sub-Saharan Africa [16]. *Fusarium* wilt was initially reported on common bean in the United States in 1929 [17]. Since then, this pathogen species has been classified as *Fusarium oxysporum* f. sp. *phaseoli* (Fop). Fungal pathogens such as *F. oxysporum* influence the production of common beans with a loss of up to 60% [18]. The pathogen invades the plant by penetrating root tissues and then spreading through the vascular system of the roots, stems, and sometimes the entire plant. This infection results in phloem blockage, internal stem discoloration, and eventual wilting. Common symptoms of (Fop) infection in common beans include stunted growth, wilting, yellowing (chlorosis), tissue death (necrosis), and, ultimately, plant death [19].

Given the substantial yield losses caused by these pathogens and their persistence in soil, the deployment of resistant cultivars remains one of the most practical and cost-effective elements of integrated disease management. Planting certified seeds of *Fusarium*-tolerant or resistant cultivars can markedly reduce disease pressure and improve productivity [20]. Nevertheless, host resistance alone is insufficient; it must be combined with classical approaches such as crop rotation, soil health improvement, sanitation, and biological or chemical interventions to achieve durable control under field conditions. Historically, common bean breeding programs have focused primarily on foliar diseases, while systematic efforts to enhance resistance against the root rot complex have been limited. Therefore, accurate identification of root rot pathogens, coupled with the screening of resistant cultivars, provides a critical foundation for sustainable management [21]. For instance, [22] evaluated 100 bean cultivars against *Fusarium* root rot and identified seven with high or moderate resistance, whereas no resistant mung bean cultivars were found, underscoring the urgent need for targeted breeding initiatives.

*Fusarium oxysporum*, documented as a vascular wilt pathogen in more than 100 plant species, has been found to cause root rot in certain cases of common bean [23,24]. *Fusarium* root rot of common bean has been severe in major production areas, and the pathogens of FRR were generally regarded as *F. solani* and *F. oxysporum* using basic morphological characteristics in previous studies [22,25]. Recent progress in molecular tools has greatly improved the early detection and accurate identification of *Fusarium* species. PCR-based assays and species-specific primers, in particular, have enhanced sensitivity and reliability compared with traditional morphology alone, thus contributing to earlier disease diagnosis and more effective management strategies [26,27]. However, the application of multilocus molecular phylogenetic analysis has led to the identification of numerous cryptic species in the *F. solani* species complex and *F. oxysporum* species complex, which have been resolved and described as new species [27,28]. Therefore, the objective of this study was to isolate the fungi associated with diseased common bean and identify the pathogenic fungal isolates using a pathogenicity test, morphological characterization, molecular phylogenetic analysis, and screening for resistance cultivars.

## 2. Materials and Methods

### 2.1. Isolation and Morphological Characterization of Fusarium spp.

A total of 50 naturally infected common bean plants showing typical root rot symptoms were collected from fields in Beni Suef, Qalyubia, Kafr El-Sheikh, and Beheira governorates in Egypt. The infected root samples were first cut into small pieces of 5 mm and washed under running tap water. They were then air-dried, surface-disinfected by dipping in a 3% sodium hypochlorite solution for 2 min, rinsed twice in sterile distilled water, and dried using sterilized filter papers. The sterilized plant pieces were transferred under aseptic conditions to 9-cm Petri dishes containing potato dextrose agar (PDA) medium. The plates were incubated at 25 ± 2 °C and visually inspected every day for 1 week. Emerging fungi were purified using single-spore isolation, as described by [29]. In which single spore suspensions were diluted, and individual germinated spores were transferred under a stereomicroscope to fresh PDA plates to establish pure cultures.

*Morphological* identification of *Fusarium* isolates was performed based on standard taxonomic keys of Leslie and Summerell [29], considering colony characteristics such as appearance on the upper and lower surfaces of culture media, pigmentation, and growth rate, as well as spore morphology, including the size and shape of macroconidia and microconidia and the presence or absence of chlamydospores.

### 2.2. Pathogenicity Test

Pathogenicity of the recovered *Fusarium* isolates was tested under greenhouse conditions at the Vegetables Disease Research Department, Plant Pathology Research Institute, Giza, Egypt. Six cultivars, Alpha, Samantha, Giza 6, Giza 12, Cambo, and Nebraska, were chosen for their agronomic importance, prevalence in Egyptian production systems, and known or suspected differences in resistance to root rot. Certified pathogen-free seeds were obtained from the Department of Vegetables Production Research, Horticultural Research Institute, Agricultural Research Center, Egypt.

Isolates were grown on potato dextrose agar for 7 days at 25 ± 2 °C. Mycelial plugs of 5 mm were transferred into bottles containing sterilized sand–sorghum medium prepared from 25 g washed sand, 75 g sorghum grains, and 80 mL distilled water. Bottles were sealed with cotton wool and aluminum foil and incubated at 25 ± 2 °C for 14 days with regular shaking to promote fungal colonization [30]. Thereafter, the colonized inoculum was mixed thoroughly into sterilized sandy loam soil at a concentration of 2.5% (*w*/*w*), which is the standard rate used for *Fusarium*–common bean pathogenicity studies [31]. Plastic pots of 20 cm diameter were filled with 1 kg of infested soil. Nine healthy, surface-sterilized seeds of each of six common bean cultivars were sown per pot. Each treatment was replicated three times. Non-infested pots, filled with sterilized soil and sown with the same cultivars, served as the negative control. The experiment was arranged in a completely randomized block design with three replicates, each consisting of three pots per cultivar. Greenhouse conditions were maintained at 25–28 °C with 60–70% relative humidity, natural photoperiod of approximately 12–13 h light per day, and regular irrigation.

### 2.3. Disease Assessment

Disease assessment of the cultivated common bean plants was evaluated at three stages:

Pre-emergence damping-off (15 days after sowing), post-emergence damping-off (30 days after sowing), and root rot severity at the flowering stage (approximately 45 days after sowing).

The pre-and post-emergence damping-off percentages were assessed as described by the method of [32] according to the following formula:

$$Pre\text{-}emergence\;damping\;off\%=\frac{n}{B}\times 100$$

where *n* = the number of non-emerged seeds and *B* = the total number of seeds sown.

$$Post\text{-}emergence\;damping\;off\%=\frac{n}{E}\times 100$$

where *n* = the number of dead plants and *E* = the total number of emerging plants.

The percentages of root rot disease incidence and the efficacy of treatments were assessed 45 days after the sowing date using the following formula:

$$Disease\;Incidence=\frac{Number\;of\;infected\;plants}{total\;number\;of\;plants\;}\times 100$$

The disease severity was evaluated 45 days after sowing, depending on the progress of symptoms, according to the method described by [33]. Lesions on roots and hypocotyls were scored on a 0–5 scale: 0 = no visible symptoms; 1 = slight brown discoloration affecting up to 20% of tissue; 2 = 21–40% of tissue showing moderate discoloration; 3 = 41–60% affected, with pronounced discoloration, root pruning, and hypocotyls collapsing under pressure; 4 = 61–80% affected, with darkly discolored hypocotyls and roots severely pruned or completely collapsed; and 5 = more than 80% of tissue affected or plants completely dead. The percentage of disease severity (DS%) was calculated according to the formula:

$$Disease\;Severity\;\%=\frac{\sum n\times r}{T\times M}\times 100$$

where *n* = number of plants in each numerical rate, *r* = rating category, *T* = total number of plants, *M* = the maximum numerical rate.

### 2.4. DNA Extraction

Genomic DNA was extracted from the five most aggressive *Fusarium* isolates selected for molecular identification. Fresh mycelial plugs (7 mm diameter) were transferred into 250 mL Erlenmeyer flasks containing 50 mL of potato dextrose broth (PDB) and incubated for 5 days at 25 °C on a rotary shaker at 100 rpm. Fungal mats were harvested by filtration through sterile cheesecloth, rinsed with sterile distilled water, and blotted dry. Approximately 1–2 g of fungal biomass was ground in liquid nitrogen, and DNA was extracted following the Dellaporta method [34] with minor modifications. In brief, powdered tissue was suspended in extraction buffer (100 mM Tris-HCl, pH 8.0, 50 mM EDTA, 500 mM NaCl, 1% SDS) and incubated at 65 °C for 10 min. An equal volume of chloroform–isoamyl alcohol (24:1) was added, mixed gently, and centrifuged to separate phases. The aqueous phase was transferred to a fresh tube, and DNA was precipitated with cold isopropanol, washed with 70% ethanol, air-dried, and dissolved in TE buffer. Subsequently, DNA concentration was measured using a NanoDrop One spectrophotometer (Thermo Fisher Scientific, Waltham, MA, USA), and integrity was confirmed by electrophoresis on 1.5% agarose gels.

### 2.5. Molecular and Phylogenetic Analysis

Molecular identification of the five most aggressive *Fusarium* isolates was performed by amplifying the translation elongation factor 1-alpha (*TEF1-α)* gene using the protocol described by [35] with slight modifications. The *TEF1-α* marker was selected for molecular identification because it is widely recognized as a standard taxonomic locus, offering high discriminatory power and reliable single-locus resolution for distinguishing closely related *Fusarium* species. The primer pair used for amplification was EF1 (5′-ATGGGTAAGGA(A/G) GACAAGAC-3′; forward) and EF2 (5′-GGA(G/A) GTACCAGT(G/C) ATCATGTT-3′; reverse), as reported by [36], which amplifies an approximately 700 bp fragment.

Polymerase chain reaction (PCR) was performed in a final volume of 25 µL consisting of 12.5 µL EmeraldAmp GT PCR Master Mix (Takara Bio Inc., Shiga, Japan), 1 µL of each primer, 2.5 µL genomic DNA template, and sterile distilled water to make up the volume. Reactions were run in an Applied Biosystems 2720 thermal cycler (Applied Biosystems, Foster City, CA, USA). The amplification program consisted of an initial denaturation at 95 °C for 2 min, followed by 35 cycles at 95 °C for 30 s, 56 °C for 40 s, and 72 °C for 1 min, with a final extension at 72 °C for 5 min. PCR products were visualized on a 1.5% agarose gel in 1× TBE buffer stained with EZ-Vision^®^ DNA dye (AMRESCO, Solon, OH, USA). Band sizes were estimated using the GeneRuler 1 kb DNA ladder (Thermo Fisher Scientific, Waltham, MA, USA).

The PCR products were purified and sequenced by Macrogen Inc. (Seoul, Republic of Korea). Raw sequence chromatograms were processed using BioEdit v7.2.5 [37] to trim low-quality regions at the 5′ and 3′ ends. The cleaned nucleotide sequences were translated using the NCBI ORF Finder to confirm the absence of internal stop codons and ensure the correctness of the open reading frame. Only high-quality, curated sequences were used in downstream analyses.

Each sequence was then submitted to the NCBI GenBank database, and corresponding accession numbers were obtained. Final sequences were deposited in the GenBank database under accession numbers PV595127–PV595131. To determine species identity, the sequences were aligned and compared to GenBank sequences using the BLASTn algorithm. Multiple sequence alignment was carried out with ClustalW v2.1, and phylogenetic analysis was performed in MEGA X v10.2.6 [38] using the neighbor-joining method with 1000 bootstrap replicates.

### 2.6. Statistical Analysis

Data were analyzed statistically using the Social Sciences (SPSS) software for Windows version 25.0. All comparisons were first subjected to a one-way analysis of variance (ANOVA) test. The significant differences among treatments means were determined with Duncan’s Multiple Range test at *p* ≤ 0.05. All values represent means of three replicates ± standard error.

## 3. Results

### 3.1. Frequency and Morphological Characterization of Fusarium Isolates

A total of 35 *Fusarium* isolates were recovered from diseased common bean plants collected from four Egyptian governorates. Qalyubia showed the highest number of isolates (20), followed by Kafr El-Sheikh (7), Beni-Suef (5), and Beheira (3) (Table 1). All isolates were purified, morphologically identified, and evaluated for their pathogenicity against six common bean cultivars (Alpha, Samantha, Giza 6, Giza 12, Cambo, and Nebraska).

**Figure 1:**
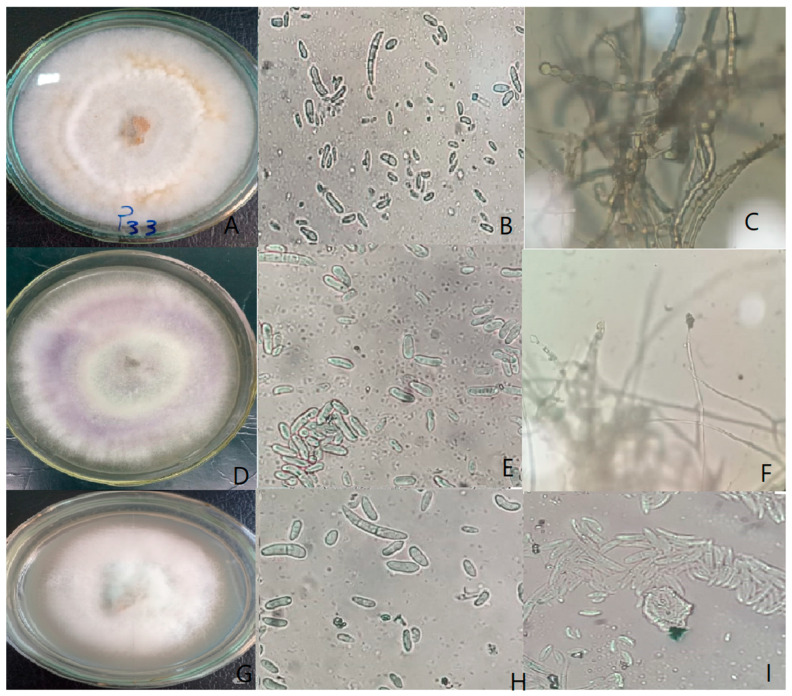
Morphological characters of the *Fusarium* spp. Isolated from common bean plants. (**A**–**C**) *F. equiseti* (isolate FP33) growth on PDA; macroconidia and microconidia; chlamydospores. (**D**–**F**) *F. oxysporum* (isolate FP26) growth on PDA; macroconidia and microconidia; microconidiophores (monophialides). (**G**–**I**) *F. solani* (isolate FP11) growth on PDA; macroconidia and microconidia; microconidia produced in sporodochia. ِAll microscopic observations were taken at 40× magnification.

The *Fusarium* isolates were morphologically characterized based on distinctive features, including colony appearance on both the upper and lower surfaces of PDA medium, pigmentation, and radial growth rate. Spore morphology, specifically the size and shape of macroconidia and microconidia, as well as the presence or absence of chlamydospores. Results indicated that colony diameters on PDA after 7 days at 25 °C ranged from 45 to 72 mm. Macroconidia length varied between 18 and 40 μm with 3–5 septa, with species differing in curvature and wall thickness: *F. equiseti* produced strongly curved, thick-walled spores with frequent chlamydospores, whereas *F. oxysporum* produced shorter, slightly curved spores. Based on these characteristics, the isolates were classified into nine *Fusarium* species: *F. proliferatum*, *F. solani*, *F. equiseti*, *F. verticillioides*, *F. semitectum*, *F. subglutinans*, *F. oxysporum*, *F. anthophilum*, and *F. tricinctum* (Table 1 and Figure 1).

The most frequent were *F. proliferatum* (8 isolates), *F. solani* (7 isolates), and *F. equiseti* (6 isolates), together representing over 60% of the total. Pathogenicity tests revealed that 13 isolates were pathogenic, including 4 *F. proliferatum*, 2 *F. oxysporum*, 2 *F. equiseti*, 2 *F. verticillioides*, 1 *F. solani*, 1 *F. subglutinans*, and 1 *F. semitectum*, while the remaining isolates were non-pathogenic (Table 1).

### 3.2. Pre-Emergence (%) Damping-Off of Fusarium Species with Different Cultivars of Common Beans

The pre-emergence damping-off varied notably among *Fusarium* isolates and common bean cultivars (Table 2). Isolate FP33 caused the highest damping-off percentages across all cultivars, reaching up to 44.44% in Alpha, Samantha, Giza 6, and Cambo, and 22.22% in Nebraska and Giza 12. Other highly pathogenic isolates included FP24, FP26, and FP21, which also produced high damping-off levels (33.33–44.44%) in Alpha, Samantha, and Giza 6, and moderate values (11.11–22.22%) in Nebraska and Giza 12. FP11 and FP17 showed moderate effects, causing up to 33.33% damping-off in Giza 6 and Cambo. In contrast, isolates such as FP1, FP18, FP2, FP8, FP10, FP14, and FP15 caused only low damping-off (≤22.22%) across most cultivars. The uninfected control plants showed no damping-off (0%).

### 3.3. Post-Emergence (%) Damping-Off of Fusarium Species with Different Cultivars of Common Beans

The post-emergence damping-off caused by *Fusarium* isolates varied among isolates and cultivars (Table 3). Isolate FP33 was the most aggressive, causing the highest damping-off percentages across all cultivars, with values ranging from 16.67% in Nebraska and Giza 12 to 25% in Alpha, Samantha, Giza 6, and Cambo. Isolates FP24, FP26, and FP21 also caused significant damping-off (14.29–25%), particularly in Alpha, Samantha, Giza 6, and Cambo. FP11 produced moderate effects (14.29–20%) across several cultivars, especially Samantha, Giza 6, and Cambo. By contrast, most isolates (FP1, FP2, FP8, FP10, FP14, FP15, FP17, and FP18) did not cause any post-emergence damping-off (0%) in any cultivar. Control plants also showed no damping-off (0%).

### 3.4. Disease Incidence Percentage on Fusarium Isolates with Different Cultivars of Common Beans

The results in Table 4 showed clear differences in disease incidence depending on both the *Fusarium* isolate and the common bean cultivar tested. The most aggressive isolates (FP33, FP24, FP26, FP21, and FP11) caused 100% disease incidence in the highly susceptible cultivars Alpha, Samantha, Giza 6, and Cambo. On the other hand, the same isolates produced lower values ranging from 25 to 33.3% on Nebraska and Giza 12, indicating partial resistance.

By contrast, isolates FP2, FP8, FP10, and FP14 showed very low incidence, ranging from 0 to 14.3%, and in some cases, no disease symptoms were observed, such as FP8 and FP14 in Nebraska. Moderate incidence, ranging from 25% to 33.3%, was recorded for FP18, FP1, FP15, and FP17, suggesting intermediate virulence. Overall, cultivar susceptibility could be ranked as follows: Giza 6, Alpha, Samantha, and Cambo were the most susceptible, while Giza 12 showed moderate resistance, and Nebraska was the most resistant cultivar.

### 3.5. Disease Severity Percentage on Fusarium Isolates with Different Cultivars of Common Beans

**Figure 2:**
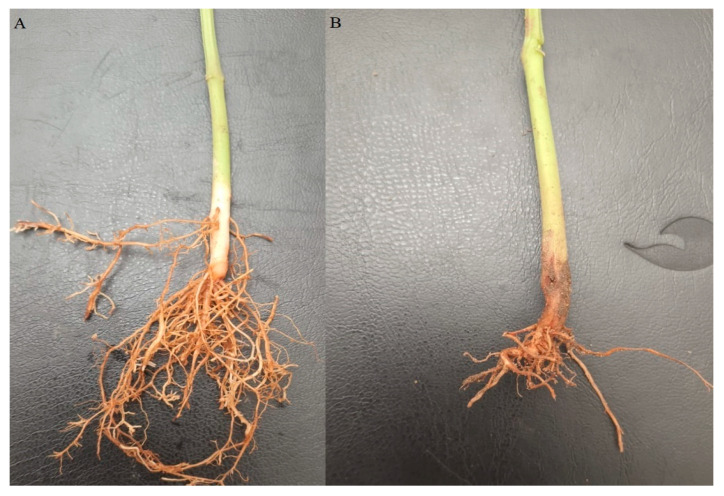
Typical symptoms of common bean root rot (**A**) uninfected control; (**B**) root of Samantha cultivar infected with *Fusarium equiseti* isolate FP33.

Disease severity results presented in Table 5 confirmed the aggressiveness of some *Fusarium* isolates. FP33 was the most virulent, causing severity values up to 90% in Giza 6 and 80% in Cambo, followed by 65% in Alpha and 70% in Samantha (Figure 2). However, its effect was much lower on Nebraska (30%) and Giza 12 (33%), which appeared as the most resistant cultivars. Similar results were observed for FP24, FP26, FP21, and FP11, which also caused high severity ranging from 60 to 85% on Alpha, Samantha, Giza 6, and Cambo, but significantly lower values of 20 to 33% on Nebraska and Giza 12.

Moderate disease severity was observed with isolates FP18, FP1, FP15, and FP17, which ranged from 25 to 33%. In contrast, the least aggressive isolates (FP2, FP8, FP10, and FP14) produced very low severity levels of 0 to 14% and, in some cases, did not induce any visible disease symptoms on certain cultivars. Overall, Giza 6 emerged as the most susceptible cultivar, followed by Alpha, Samantha, and Cambo, while Nebraska and Giza 12 demonstrated the greatest resistance.

### 3.6. Molecular Identification and Phylogenetic Analysis

**Figure 3:**
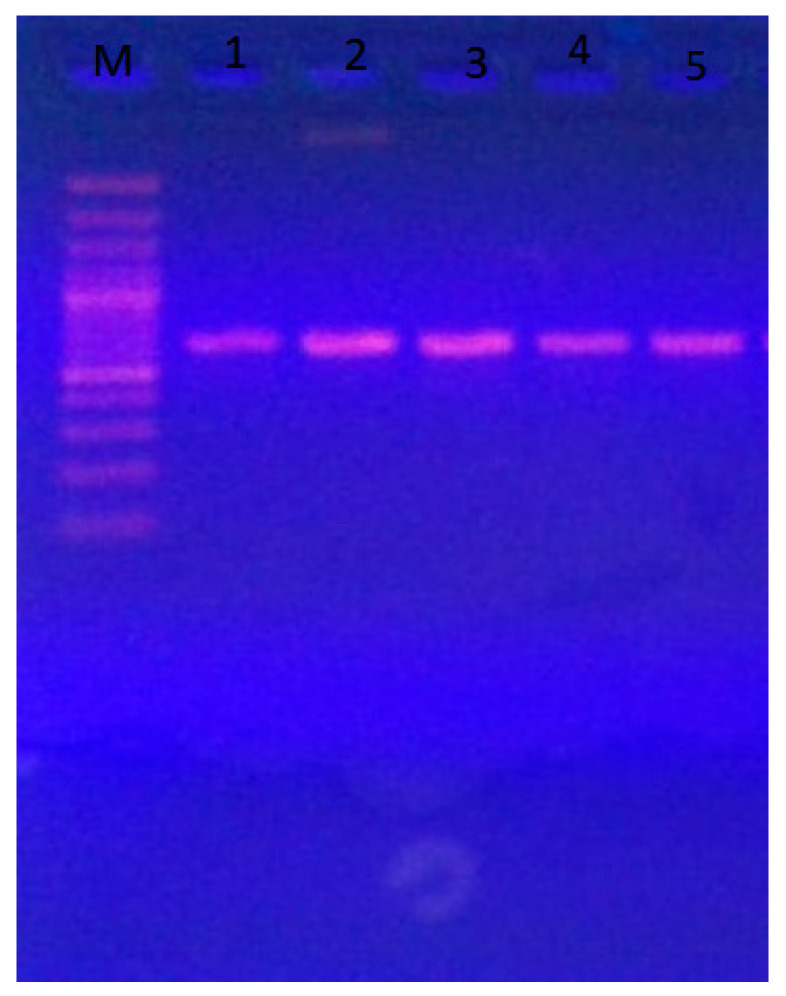
Agarose gel electrophoresis of PCR-amplified *TEF1-α* gene (~700 bp) from the five most aggressive *Fusarium* isolates associated with common bean root rot. Lane M: GeneRuler 1 kb DNA marker (Thermo Fisher Scientific); lanes 1–5: FP11, FP21, FP24, FP26, and FP33: respective *Fusarium* isolates.

The *TEF1-α* gene region (~700 bp) was successfully amplified from the five most aggressive *Fusarium* isolates associated with common bean root rot. Clear single bands were obtained for all isolates on agarose gel (Figure 3), confirming successful PCR amplification. The purified PCR products were then sequenced, and the resulting nucleotide sequences were analyzed using the BLASTn algorithm against the NCBI GenBank database.

Specifically, FP33 (Kafr El-Sheikh) and FP24 (Beni-Suef) were identified as *Fusarium equiseti*, FP26 (Kafr El-Sheikh) and FP21 (Beni-Suef) as *Fusarium oxysporum*, and FP11 (Qalyubia) as *Fusarium solani*. The percentage identity ranged from 96.45% to 99.69%, query coverage from 89% to 94%, and all E-values were 0.0 (Table 6). All sequences were deposited in GenBank under accession numbers PV595127–PV595131.

**Figure 4:**
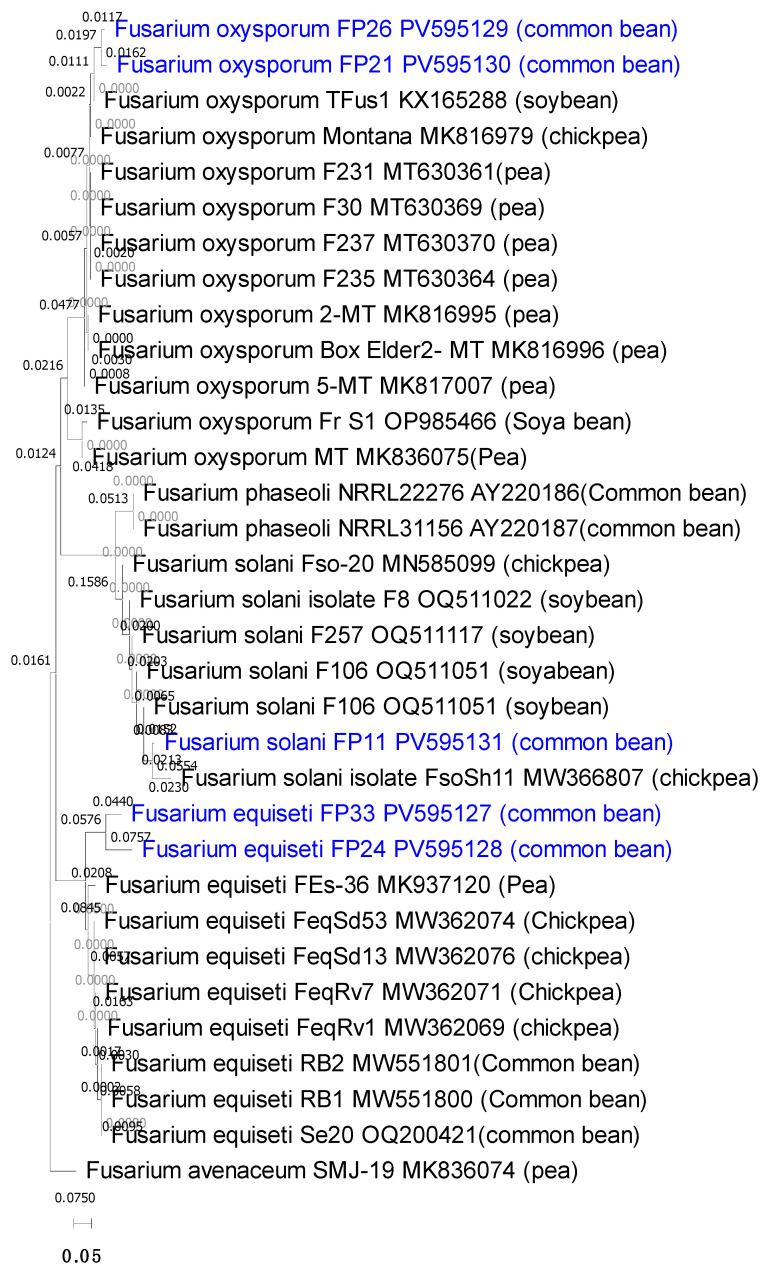
A neighbor-joining phylogenetic tree based on *TEF-1α* gene sequences illustrating the relationships between the five most potent *Fusarium* isolates and their closely related members from the NCBI database. The tree was constructed using 1000 replications of resampled datasets. *Fusarium avenaceum* included as outgroup.

Multiple sequence alignment using ClustalW and a neighbor-joining phylogenetic tree generated in MEGA X with 1000 bootstrap replicates grouped the isolates into three well-supported clades corresponding to *F. equiseti*, *F. oxysporum*, and *F. solani* (Figure 4). The common bean associated isolates clustered closely with *Fusarium* sequences previously reported from other legumes, including soybean, chickpea, and pea (Table 7). Specifically, the *F. oxysporum* isolates formed a clade with sequences from pea and soybean roots and stems, *F. solani* clustered with chickpea and soybean isolates from the USA, and *F. equiseti* clustered with sequences from chickpea, pea, and previously reported common bean isolates from Iran. confirming their taxonomic placement and suggesting potential cross-host pathogenic relationships.

## 4. Discussion

*Fusarium* species are among the most important soil-borne pathogens responsible for severe losses in common bean production worldwide. The present study provides an integrated assessment of the diversity, pathogenicity, and genetic variability of *Fusarium* isolates associated with common bean root rot in Egypt, combining morphological characterization, greenhouse pathogenicity assays, and molecular identification. This multifaceted approach allowed not only the confirmation of species identity but also the exploration of their pathogenic variability and implications for disease management.

Out of 35 isolates recovered from symptomatic roots, nine *Fusarium* species were identified: *F. proliferatum*, *F. solani*, *F. equiseti*, *F. verticillioides*, *F. semitectum*, *F. subglutinans*, *F. oxysporum*, *F. anthophilum*, and *F. tricinctum*. This high diversity indicates a complex disease etiology in Egyptian fields, consistent with previous reports documenting the occurrence of multiple *Fusarium* species in common bean production systems worldwide [18,26,47]. For instance, several *Fusarium* taxa were isolated from beans in Iran [47], in Michigan, United States [48], and with specific species such as *F. cuneirostrum* confirmed in Canada [49], highlighting their global prevalence and adaptability.

Greenhouse assays revealed significant heterogeneity in pathogenicity. Isolates FP33, FP24, FP26, FP21, and FP11 were highly virulent, inducing severe pre- and post-emergence damping-off, high disease incidence, and extensive root rot lesions. By contrast, FP8, FP14, FP2, and FP10 were either non-pathogenic or weakly virulent, while FP18, FP1, FP15, and FP17 displayed intermediate aggressiveness. This gradient of pathogenicity reflects the well-documented heterogeneity of *Fusarium* populations, where aggressive and weakly pathogenic strains coexist within the same field [50,51,52]. Such variability represents an adaptive advantage, enabling persistence under diverse soil and climatic conditions.

The observed pathogenic spectrum (37% of isolates being highly aggressive) aligns with previous studies reporting approximately 40% of *Fusarium* strains as strongly pathogenic on bean seedlings [21]. These findings emphasize that effective disease management strategies must account for the coexistence of diverse pathogenic races, as single-gene resistance is unlikely to provide durable protection.

Marked differences in cultivar susceptibility were detected. Giza 6 and Cambo were highly susceptible, exhibiting complete mortality under infection with the most aggressive isolates, while Alpha and Samantha displayed moderate susceptibility. In contrast, Nebraska and Giza 12 consistently showed high resistance, with significantly reduced disease incidence and severity. Typical symptoms included seedling yellowing, wilting, root necrosis, and stunted growth, consistent with descriptions of FRR symptomatology [25]. These results corroborate earlier reports that resistance to FRR is quantitative rather than absolute, relying on polygenic defense mechanisms [53,54,55]. Proposed mechanisms include lignification of root tissues, enhanced peroxidase and polyphenol oxidase activity, and accumulation of antifungal metabolites, all of which limit pathogen colonization [56]. The partial resistance demonstrated by Nebraska and Giza 12 thus represents an exploitable trait in breeding programs. Importantly, the strong susceptibility of Giza 6 highlights the vulnerability of widely cultivated Egyptian varieties, underscoring the urgent need for resistant alternatives.

Disease incidence and severity data provided complementary insights into isolate–host interactions. FP33 and FP24 consistently produced the highest values, causing up to 100% incidence and 90% severity in Giza 6 and Cambo. Conversely, Nebraska and Giza 12 showed strong resistance, with incidence below 35% and severity under 30% even when inoculated with the most aggressive isolates. Such variability underscores the importance of host genotype in modulating disease outcomes. Similar quantitative resistance patterns were reported by [53], where resistant cultivars restricted lesion expansion, and by [54], who emphasized resistance in reducing disease progression.

Molecular characterization using the *TEF1-α* gene marker provided reliable confirmation of isolate identities. BLASTn analysis showed high sequence homology with reference *Fusarium* species, with identities ranging from 96.45% to 99.69%, query coverage between 89% and 94%, and all E-values equal to 0.0, confirming the robustness of the molecular approach. The five most virulent isolates were identified as *F. equiseti* (FP33, FP24), *F. oxysporum* (FP26, FP21), and *F. solani* (FP11), consistent with their pathogenic profiles. *TEF1-α* is considered a highly informative marker for *Fusarium* taxonomy due to its discriminatory power at the species level [57,58]. Ref. [59] reported that *Fusarium* species involved in bean root rot in Zanjan province are *F. solani*, *F. equiseti*, *F. oxysporum*, *F. crookwellense*, *F. acuminatum*, and *F. sambucinum*. However, the predominance of *F. equiseti* in our collection is noteworthy, as this species is often reported as secondary compared with the more widely studied *F. solani* and *F. oxysporum* [52,60]. This novel finding emphasizes the shifting dynamics of pathogen populations under Egyptian agroecological conditions and warrants further investigation.

Phylogenetic analysis clustered the isolates into three distinct clades corresponding to *F. equiseti*, *F. oxysporum*, and *F. solani*, with strong bootstrap support. Importantly, Egyptian isolates grouped with *Fusarium* strains previously reported from other legumes, including soybean, chickpea, and pea. For example, *F. oxysporum* isolates clustered with pea and soybean sequences from Europe and the USA, while *F. equiseti* grouped with chickpea and common bean isolates from Iran and Morocco. This clustering highlights intra-specific diversity and suggests potential cross-host pathogenicity, raising epidemiological concerns about the capacity of *Fusarium* strains to adapt to multiple legume hosts. Similar observations of cross-host clustering and intra-specific variability have been reported previously, highlighting the adaptability of these pathogens across different legumes [61].

The predominance of *F. equiseti* in Egypt may be linked to specific agroecological conditions. Egypt’s hot, dry climate interspersed with periods of irrigation, creates fluctuating soil moisture and temperature regimes favorable for diverse *Fusarium* species. *F. equiseti*, in particular, has been reported to thrive in semi-arid environments and may outcompete other *Fusarium* taxa under such conditions [62]. Moreover, continuous bean cultivation and limited crop rotation in Egyptian fields could contribute to pathogen build-up and selection of highly aggressive strains. Understanding these ecological drivers is critical for designing targeted disease management practices.

The genetic variability observed within and among *Fusarium* species also has epidemiological implications. The coexistence of diverse lineages within Egyptian fields suggests multiple sources of inoculum and possible introduction through seed trade. This diversity enhances the adaptive potential of pathogen populations, increasing the risk of resistance breakdown if single-gene resistance is deployed [63].

Looking ahead, the findings of this study provide a foundation for designing sustainable management strategies against *Fusarium* root rot in common bean. Breeding programs should prioritize the incorporation of quantitative resistance traits, as demonstrated by Nebraska and Giza 12, to achieve durable resistance across multiple *Fusarium* species. Molecular tools such as *TEF1-α* should be further exploited for rapid pathogen surveillance and to monitor the emergence of new virulent strains. Additionally, biological control using antagonistic microorganisms and plant growth-promoting rhizobacteria represents an eco-friendly alternative that can complement host resistance. From an agroecological perspective, integrating crop rotation, soil amendments, and optimized irrigation practices could suppress *Fusarium* inoculum in fields. Finally, mechanistic studies focusing on host defense pathways, including secondary metabolite biosynthesis and systemic resistance induction, are needed to clarify the molecular basis of partial resistance. Together, these strategies will enhance integrated disease management, safeguard bean productivity, and contribute to sustainable legume cultivation under Egyptian and global conditions.

## 5. Conclusions

This study highlights the significant role of Fusarium species in causing root rot in common bean, identifying *F. equiseti*, *F. oxysporum*, and *F. solani* as the primary pathogens responsible for disease development. The findings demonstrated considerable genetic variability among the isolates, emphasizing the complexity of root rot etiology in common bean fields in Egypt. The high virulence of specific isolates, particularly *F. equiseti* (FP33 and FP24), underscores the need for targeted disease management strategies. The results also highlight the varying resistance levels among common bean cultivars, suggesting that breeding for genetic resistance remains a crucial approach to mitigate yield losses. Future studies should focus on exploring host–pathogen interactions, screening for resistant genotypes, and developing integrated disease management strategies that combine resistant cultivars. Overall, this research contributes to a better understanding of Fusarium diversity and its impact on common bean production. The integration of molecular tools in pathogen identification can enhance disease diagnosis and guide the development of effective and sustainable control measures to improve common bean productivity and food security.

## Figures and Tables

**Table 1:** *Fusarium* species isolated from common bean and their pathogenicity on common bean plants.

Isolate Code	Governorate	*Fusarium* spp.	Pathogenic Reaction
FP1	Qalyubia	*F. proliferatum*	Pathogenic
FP2	Qalyubia	*F. proliferatum*	Pathogenic
FP3	Qalyubia	*F. equiseti*	Non-pathogenic
FP4	Qalyubia	*F. verticillioides*	Non-pathogenic
FP5	Qalyubia	*F. semitectum*	Non-pathogenic
FP6	Qalyubia	*F. solani*	Non-pathogenic
FP7	Qalyubia	*F. proliferatum*	Non-pathogenic
FP8	Qalyubia	*F. proliferatum*	Pathogenic
FP9	Qalyubia	*F. solani*	Non-pathogenic
FP10	Qalyubia	*F. proliferatum*	Pathogenic
FP11	Qalyubia	*F. solani*	Pathogenic
FP12	Qalyubia	*F. equiseti*	Non-pathogenic
FP13	Qalyubia	*F. verticillioides*	Non-pathogenic
FP14	Qalyubia	*F. subglutinans*	Pathogenic
FP15	Qalyubia	*F. semitectum*	Pathogenic
FP16	Qalyubia	*F. solani*	Non-pathogenic
FP17	Qalyubia	*F. verticillioides*	Pathogenic
FP18	Kafr-elsheikh	*F. verticillioides*	Pathogenic
FP19	Kafr-elsheikh	*F. subglutinans*	Non-pathogenic
FP20	Kafr-elsheikh	*F. semitectum*	Non-pathogenic
FP21	Beni-Suef	*F. oxysporum*	Pathogenic
FP22	Beni-Suef	*F. subglutinans*	Non-pathogenic
FP23	Beni-Suef	*F. solani*	Non-pathogenic
FP24	Beni-Suef	*F. equiseti*	Pathogenic
FP25	Beni-Suef	*F. tricinctum*	Non-pathogenic
FP26	Kafr-elsheikh	*F. oxysporum*	Pathogenic
FP27	Qalyubia	*F. anthophilum*	Non-pathogenic
FP28	Qalyubia	*F. semitectum*	Non-pathogenic
FP29	Qalyubia	*F. equiseti*	Non-pathogenic
FP30	Baheira	*F. oxysporum*	Non-pathogenic
FP31	Kafr-elsheikh	*F. solani*	Non-pathogenic
FP32	Kafr-elsheikh	*F. verticillioides*	Non-pathogenic
FP33	Kafr-elsheikh	*F. equiseti*	Pathogenic
FP34	Beheira	*F. solani*	Non-pathogenic
FP35	Baheira	*F. equiseti*	Non-pathogenic

**Table 2:** Pre-emergence (%) damping-off of common bean cultivars inoculated with different *Fusarium* isolates.

		Common Bean Cultivars				
Isolate code	Alpha	Samantha	Nebraska	Giza6	Giza 12	Cambo
FP18	11.11 ± 0.64 ^d^	11.11 ± 1.28 ^d^	11.11 ± 1.28 ^d^	22.22 ± 1.28 ^c^	11.11 ± 1.92 ^d^	22.22 ± 2.95 ^c^
FP33	44.44 ± 2.57 ^a^	44.44 ± 5.13 ^a^	22.22 ± 2.57 ^c^	44.44 ± 2.57 ^a^	22.22 ± 3.85 ^c^	44.44 ± 5.9 ^a^
FP24	33.33 ± 1.92 ^b^	33.33 ± 3.85 ^b^	11.11 ± 1.28 ^d^	44.44 ± 2.57 ^a^	22.22 ± 3.85 ^c^	44.44 ± 5.9 ^a^
FP26	33.33 ± 1.92 ^b^	33.33 ± 3.85 ^b^	11.11 ± 1.28 ^d^	44.44 ± 2.57 ^a^	22.22 ± 3.85 ^c^	44.44 ± 5.9 ^a^
FP1	11.11 ± 0.64 ^d^	22.22 ± 2.57 ^c^	11.11 ± 1.28 ^d^	22.22 ± 1.28 ^c^	11.11 ± 1.92 ^d^	22.22 ± 2.95 ^c^
FP21	33.33 ± 1.92 ^b^	33.33 ± 3.85 ^b^	11.11 ± 1.28 ^d^	44.44 ± 2.57 ^a^	22.22 ± 3.85 ^c^	44.44 ± 5.9 ^a^
FP2	11.11 ± 0.64 ^d^	11.11 ± 1.28 ^d^	11.11 ± 1.28 ^d^	22.22 ± 1.28 ^c^	11.11 ± 1.92 ^d^	22.22 ± 2.95 ^c^
FP8	11.11 ± 0.64 ^d^	11.11 ± 1.28 ^d^	11.11 ± 1.28 ^d^	22.22 ± 1.28 ^c^	11.11 ± 1.92 ^d^	22.22 ± 2.95 ^c^
FP10	11.11 ± 0.64 ^d^	11.11 ± 1.28 ^d^	11.11 ± 1.28 ^d^	22.22 ± 1.28 ^c^	11.11 ± 1.92 ^d^	22.22 ± 2.95 ^c^
FP11	22.22 ± 1.28 ^c^	22.22 ± 2.57 ^c^	11.11 ± 1.28 ^d^	33.33 ± 1.92 ^b^	11.11 ± 1.92 ^d^	33.33 ± 4.43 ^b^
FP17	22.22 ± 1.28 ^c^	22.22 ± 2.57 ^c^	11.11 ± 1.28 ^d^	33.33 ± 1.92 ^b^	11.11 ± 1.92 ^d^	33.33 ± 4.43 ^b^
FP15	11.11 ± 0.64 ^d^	22.22 ± 2.57 ^c^	11.11 ± 1.28 ^d^	22.22 ± 1.28 ^c^	11.11 ± 1.92 ^d^	22.22 ± 2.95 ^c^
FP14	11.11 ± 0.64 ^d^	11.11 ± 1.28 ^d^	11.11 ± 1.28 ^d^	22.22 ± 1.28 ^c^	11.11 ± 1.92 ^d^	22.22 ± 2.95 ^c^
Control	0 ± 0 ^e^	0 ± 0 ^e^	0 ± 0 ^e^	0 ± 0 ^e^	0 ± 0 ^e^	0 ± 0 ^e^

Means within a column ± standard error followed by different letters (a, b, c, d, e) indicate significant differences among treatments according to Duncan’s multiple range tests at *p* ≤ 0.05. Values were means of three replicates for each treatment as well as the control.

**Table 3:** Post-emergence (%) damping-off of common bean cultivars inoculated with different *Fusarium* isolates.

		Common Bean Cultivars				
Isolate Code	Alpha	Samantha	Nebraska	Giza6	Giza 12	Cambo
FP18	0 ± 0 ^d^	0 ± 0 ^d^	0 ± 0 ^d^	0 ± 0 ^d^	0 ± 0 ^d^	0 ± 0 ^d^
FP33	25 ± 1.44 ^a^	25 ± 2.89 ^a^	16.67 ± 1.92 ^b^	25 ± 1.44 ^a^	16.67 ± 2.89 ^b^	25 ± 3.32 ^a^
FP24	20 ± 1.15 ^b^	20 ± 2.31 ^b^	14.29 ± 1.65 ^c^	25 ± 1.44 ^a^	16.67 ± 2.89 ^b^	25 ± 3.32 ^a^
FP26	20 ± 1.15 ^b^	20 ± 2.31 ^b^	14.29 ± 1.65 ^c^	25 ± 1.44 ^a^	16.67 ± 2.89 ^b^	25 ± 3.32 ^a^
FP1	0 ± 0 ^d^	0 ± 0 ^d^	0 ± 0 ^d^	0 ± 0 ^d^	0 ± 0 ^d^	0 ± 0 ^d^
FP21	20 ± 1.15 ^b^	20 ± 2.31 ^b^	14.29 ± 1.65 ^c^	25 ± 1.44 ^a^	16.67 ± 2.89 ^b^	25 ± 3.32 ^a^
FP2	0 ± 0 ^d^	0 ± 0 ^d^	0 ± 0 ^d^	0 ± 0 ^d^	0 ± 0 ^d^	0 ± 0 ^d^
FP8	0 ± 0 ^d^	0 ± 0 ^d^	0 ± 0 ^d^	0 ± 0 ^d^	0 ± 0 ^d^	0 ± 0 ^d^
FP10	0 ± 0 ^d^	0 ± 0 ^d^	0 ± 0 ^d^	0 ± 0 ^d^	0 ± 0 ^d^	0 ± 0 ^d^
FP11	16.67 ± 0.96 ^b^	16.67 ± 1.92 ^b^	14.29 ± 1.65 ^c^	20 ± 1.15 ^b^	14.29 ± 2.48 ^c^	20 ± 2.66 ^b^
FP17	0 ± 0 ^d^	0 ± 0 ^d^	0 ± 0 ^d^	0 ± 0 ^d^	0 ± 0 ^d^	0 ± 0 ^d^
FP15	0 ± 0 ^d^	0 ± 0 ^d^	0 ± 0 ^d^	0 ± 0 ^d^	0 ± 0 ^d^	0 ± 0 ^d^
FP14	0 ± 0 ^d^	0 ± 0 ^d^	0 ± 0 ^d^	0 ± 0 ^d^	0 ± 0 ^d^	0 ± 0 ^d^
Control	0 ± 0 ^d^	0 ± 0 ^d^	0 ± 0 ^d^	0 ± 0 ^d^	0 ± 0 ^d^	0 ± 0 ^d^

Means within a column ± standard error followed by different letters (a, b, c, d) indicate significant differences among treatments according to Duncan’s multiple range tests at *p* ≤ 0.05. Values were means of three replicates for each treatment as well as the control.

**Table 4:** Disease incidence % of *Fusarium* isolates against different cultivars of common beans.

		Common Bean Cultivars				
Isolate Code	Alpha	Samantha	Nebraska	Giza6	Giza 12	Cambo
FP18	25 ± 0.72 ^c^	25 ± 0.43 ^c^	25 ± 1.37 ^c^	28.57 ± 2.47 ^b^	25 ± 2.89 ^c^	28.57 ± 1.65 ^b^
FP33	100 ± 0 ^a^	100 ± 0 ^a^	33.33 ± 1.83 ^b^	100 ± 0 ^a^	33.33 ± 3.85 ^b^	100 ± 0 ^a^
FP24	100 ± 0 ^a^	100 ± 0 ^a^	28.57 ± 1.57 ^b^	100 ± 0 ^a^	33.33 ± 3.85 ^b^	100 ± 0 ^a^
FP26	100 ± 0 ^a^	100 ± 0 ^a^	28.57 ± 1.57 ^b^	100 ± 0 ^a^	33.33 ± 3.85 ^b^	100 ± 0 ^a^
FP1	25 ± 0.72 ^c^	28.57 ± 0.49 ^b^	25 ± 1.37 ^c^	28.57 ± 2.47 ^b^	25 ± 2.89 ^c^	28.57 ± 1.65 ^b^
FP21	100 ± 0 ^a^	100 ± 0 ^a^	28.57 ± 1.57 ^b^	100 ± 0 ^a^	33.33 ± 3.85 ^b^	100 ± 0 ^a^
FP2	12.5 ± 0.36 ^c^	12.5 ± 0.22 ^c^	12.5 ± 0.69 ^c^	14.29 ± 1.24 ^c^	12.5 ± 1.44 ^c^	14.29 ± 0.83 ^c^
FP8	12.5 ± 0.36 ^c^	12.5 ± 0.22 ^c^	0 ± 0 ^d^	14.29 ± 1.24 ^c^	0 ± 0 ^d^	14.29 ± 0.83 ^c^
FP10	12.5 ± 0.36 ^c^	12.5 ± 0.22 ^c^	12.5 ± 0.69 ^c^	14.29 ± 1.24 ^c^	12.5 ± 1.44 ^c^	14.29 ± 0.83 ^c^
FP11	100 ± 0 ^a^	100 ± 0 ^a^	25 ± 1.37 ^c^	100 ± 0 ^a^	25 ± 2.89 ^c^	100 ± 0 ^a^
FP17	28.57 ± 0.82 ^b^	28.57 ± 0.49 ^b^	25 ± 1.37 ^c^	33.33 ± 2.89 ^b^	25 ± 2.89 ^c^	33.33 ± 1.92 ^b^
FP15	25 ± 0.72 ^c^	28.57 ± 0.49 ^b^	25 ± 1.37 ^c^	28.57 ± 2.47 ^b^	25 ± 2.89 ^c^	28.57 ± 1.65 ^b^
FP14	12.5 ± 0.36 ^c^	12.5 ± 0.22 ^c^	0 ± 0 ^d^	14.29 ± 1.24 ^c^	12.5 ± 1.44 ^c^	14.29 ± 0.83 ^c^
Control	0 ± 0 ^d^	0 ± 0 ^d^	0 ± 0 ^d^	0 ± 0 ^d^	0 ± 0 ^d^	0 ± 0 ^d^

Means within a column ± standard error followed by different letters (a, b, c, d) indicate significant differences among treatments according to Duncan’s multiple range tests at *p* ≤ 0.05. Values were means of three replicates for each treatment as well as the control.

**Table 5:** Disease severity % of *Fusarium* isolates against different cultivars of common beans.

		Common Bean Cultivars				
Isolate Code	Alpha	Samantha	Nebraska	Giza6	Giza 12	Cambo
FP18	25 ± 2.89 ^c^	25 ± 1.15 ^c^	20 ± 1.27 ^d^	25.71 ± 3.27 ^c^	22.5 ± 1.69 ^d^	25.71 ± 1.48 ^c^
FP33	65 ± 7.51 ^a^	70 ± 3.23 ^a^	30 ± 1.91 ^c^	90 ± 11.43 ^a^	33.33 ± 2.5 ^c^	80 ± 4.62 ^a^
FP24	64 ± 7.39 ^a^	68 ± 3.14 ^a^	28.57 ± 1.81 ^c^	85 ± 10.8 ^a^	33.33 ± 2.5 ^c^	75 ± 4.33 ^a^
FP26	60 ± 6.93 ^b^	64 ± 2.96 ^a^	25.71 ± 1.63 ^c^	80 ± 10.16 ^a^	25.71 ± 1.93 ^c^	70 ± 4.04 ^a^
FP1	25 ± 2.89 ^c^	28.57 ± 1.32 ^c^	22.5 ± 1.43 ^d^	28.57 ± 3.63 ^c^	25 ± 1.88 ^c^	25.71 ± 1.48 ^c^
FP21	56 ± 6.47 ^b^	60 ± 2.77 ^b^	22.86 ± 1.45 ^d^	75 ± 9.53 ^a^	25.71 ± 1.93 ^c^	65 ± 3.75 ^a^
FP2	10 ± 1.15 ^d^	12.5 ± 0.58 ^d^	7.5 ± 0.48 ^e^	14.29 ± 1.82 ^d^	10 ± 0.75 ^d^	11.43 ± 0.66 ^d^
FP8	7.5 ± 0.87 ^e^	10 ± 0.46 ^d^	0 ± 0 ^e^	11.43 ± 1.45 ^d^	0 ± 0 ^e^	8.57 ± 0.49 ^c^
FP10	12.5 ± 1.44 ^d^	12.5 ± 0.58 ^d^	10 ± 0.64 ^d^	14.29 ± 1.82 ^d^	10 ± 0.75 ^d^	14.29 ± 0.83 ^d^
FP11	51.43 ± 5.94 ^b^	60 ± 2.77 ^b^	22.5 ± 1.43 ^d^	70 ± 8.89 ^a^	25 ± 1.88 ^c^	63.33 ± 3.66 ^a^
FP17	28.57 ± 3.3 ^c^	28.57 ± 1.32 ^c^	22.5 ± 1.43 ^d^	30 ± 3.81 ^c^	25 ± 1.88 ^c^	30 ± 1.73 ^c^
FP15	25 ± 2.89 ^c^	28.57 ± 1.32 ^c^	22.5 ± 1.43 ^d^	28.57 ± 3.63 ^c^	25 ± 1.88 ^c^	28.57 ± 1.65 ^c^
FP14	10 ± 1.15 ^d^	10 ± 0.46 ^d^	0 ± 0 ^e^	11.43 ± 1.45 ^d^	7.5 ± 0.56 ^e^	11.43 ± 0.66 ^d^
Control	0 ± 0 ^e^	0 ± 0 ^e^	0 ± 0 ^e^	0 ± 0 ^e^	0 ± 0 ^e^	0 ± 0 ^e^

Means within a column ± standard error followed by different letters (a, b, c, d, e) indicate significant differences among treatments according to Duncan’s multiple range tests at *p* ≤ 0.05. Values were means of three replicates for each treatment as well as the control.

**Table 6:** The accession number of the most aggressive isolates that were submitted to and retrieved from NCBI database.

Isolate Code	Fusarium Species	Governorate	Closest GenBank Match	Identity (%)	Query Coverage (%)	E-Value	GenBank Accession Number
FP33	*Fusarium equiseti*	Kafr-elsheikh	MK937120	96.45	89%	0.0	PV595127
FP24	*Fusarium equiseti*	Beni-Suef	MW362076	97.13	94%	0.0	PV595128
FP26	*Fusarium oxysporum*	Kafr-elsheikh	KX165288	98.79	93%	0.0	PV595129
FP21	*Fusarium oxysporum*	Beni-Suef	MT 630364	99.69	93%	0.0	PV595130
FP11	*Fusarium solani*	Qalyubia	OQ511051	99.42	91%	0.0	PV595131

**Table 7:** *Fusarium* species and their genBank accession numbers related to other research used in this study.

Species from Isolate	Origin Plant	Isolation Source	References	Country	GenBank Accession Number
*Fusarium oxysporum*	pea	Root	[39]	United Kingdom	MT 630361
*Fusarium oxysporum*	pea	Root	[39]	United Kingdom	MT 630369
*Fusarium oxysporum*	pea	Root	[39]	United Kingdom	MT 630370
*Fusarium oxysporum*	pea	Root	[39]	United Kingdom	MT 630364
*Fusarium oxysporum*	soybean	Stem	[40]	Croatia	KX165288
*Fusarium oxysporum*	chickpea	Root	[41]	Gallatin, Montana	MK816979
*Fusarium oxysporum*	pea	Root	[41]	Daniels, Montana USA	MK816995
*Fusarium oxysporum*	pea	Root	[41]	Sheridan, USA	MK816996
*Fusarium oxysporum*	pea	Root	[41]	Daniels, Montana USA	MK817007
*Fusarium oxysporum*	soybean	Seed	[42]	Poland	OP985466
*Fusarium oxysporum*	pea	Root	[41]	Daniels, Montana USA	MK836075
*Fusarium phaseoli*	Common bean	Root	[43]	USA	AY220186
*Fusarium phaseoli*	Common bean	Root	[43]	USA	AY220187
*Fusarium solani*	chickpea	Root	[43]	Gallatin, Montana USA	MN585099
*Fusarium solani*	soybean	Root	[44]	Pennsylvania USA	OQ511117
*Fusarium solani*	soybean	Root	[44]	Pennsylvania USA	OQ511022
*Fusarium solani*	soybean	Root	[44]	Pennsylvania, USA	OQ511051
*Fusarium solani*	chickpea	Root	[45]	Montana, USA	MW366807
*Fusarium equiseti*	pea	seed	[41]	USA	MK937120
*Fusarium equiseti*	chickpea	Root	[45]	USA	MW362071
*Fusarium equiseti*	chickpea	Root	[45]	USA	MW362074
*Fusarium equiseti*	chickpea	Root	[45]	USA	MW362076
*Fusarium equiseti*	chickpea	Root	[45]	USA	MW362069
*Fusarium equiseti*	Common bean	Root	[46]	Khomein, Iran	MW551801
*Fusarium equiseti*	Common bean	Root	[46]	Khomein, Iran	MW551800
*Fusarium equiseti*	Common bean	Root	[47]	Selseleh	OQ200421

## Data Availability

The original contributions presented in this study are included in the article. Further inquiries can be directed to the corresponding authors.
